# The Rapid Emergence of Stimulus Specific Perceptual Learning

**DOI:** 10.3389/fpsyg.2012.00226

**Published:** 2012-07-05

**Authors:** Zahra Hussain, Paul V. McGraw, Allison B. Sekuler, Patrick J. Bennett

**Affiliations:** ^1^School of Psychology, University of NottinghamNottingham, UK; ^2^Department of Psychology, Neuroscience and Behaviour, McMaster UniversityHamilton, ON, Canada; ^3^Centre for Vision Research, York UniversityToronto, ON, Canada

**Keywords:** perceptual learning, identification, generalization, transfer, specificity, time course, consolidation

## Abstract

Is stimulus specific perceptual learning the result of extended practice or does it emerge early in the time course of learning? We examined this issue by manipulating the amount of practice given on a face identification task on Day 1, and altering the familiarity of stimuli on Day 2. We found that a small number of trials was sufficient to produce stimulus specific perceptual learning of faces: on Day 2, response accuracy decreased by the same amount for novel stimuli regardless of whether observers practiced 105 or 840 trials on Day 1. Current models of learning assume early procedural improvements followed by late stimulus specific gains. Our results show that stimulus specific and procedural improvements are distributed throughout the time course of learning.

## Introduction

Improvements in perceptual and motor skills often follow a time course comprising steep early gains, followed by gradual increases in performance that accumulate over the course of several hundreds or thousands of trials (Poggio et al., 1992; Karni and Sagi, 1993; Recanzone et al., 1993; Ahissar and Hochstein, 1997; Karni et al., 1998; Wright and Fitzgerald, 2001; Hussain et al., 2009a; Ortiz and Wright, 2009; Agus et al., 2010). Rapid improvements within the first few trials are attributed to learning of task demands and general aspects of the procedure, termed task-related, conceptual, or procedural learning (Recanzone et al., 1993; Karni and Bertini, 1997; Karni et al., 1998; Wright and Fitzgerald, 2001). Stimulus specific learning – the proportion of learning that is specific to the trained stimulus attributes, retinal location, or eye (Fiorentini and Berardi, 1980; Ball and Sekuler, 1987; Poggio et al., 1992; Karni and Sagi, 1993; Ahissar and Hochstein, 1997; Hawkey et al., 2004; Aberg et al., 2009; Hussain et al., 2009a; Ortiz and Wright, 2009; Jeter et al., 2010) – is thought to be a slower process occurring later, after the cumulation of several hundred trials or more, and is associated with different mechanisms of plasticity than those involved in task-related learning (Karni and Bertini, 1997; Zhaoping et al., 2003; Law and Gold, 2009). By this view, the early and late phases of perceptual learning are associated with distinct types of learning.

On the other hand, it is known that for a variety of tasks, learning approximates a power or exponential form: when performance is plotted against log number of practice trials, no clear discontinuity in performance emerges (Dosher and Lu, 2005, 2007; Hussain et al., 2009b). In other words, performance is a linear function of the log number of practice trials, which could be viewed as inconsistent with the idea that learning consists of qualitatively distinct, early and late stages. From this perspective, stimulus specificity of learning is predicted to emerge both early and late in practice. The most straightforward method of establishing whether specificity emerges early is to vary the amount of practice before subjects transfer to a different stimulus or task. One study in the visual domain (Jeter et al., 2010) explicitly used this method to show early generalization, followed by late specificity of learning of an orientation discrimination task. Aberg et al. (2009) reported a similar finding in a study examining the minimum amount of practice needed to improve Chevron discrimination. In the auditory domain, however, stimulus-related learning has been reported to emerge early (Hawkey et al., 2004), and one study has shown that late trials contribute to generalizable improvements (Wright et al., 2010).

Previous studies have shown that robust, long-lasting, stimulus specific improvements are found on face identification after large amounts of practice (Hussain et al., 2011), and that small amounts are sufficient to raise performance to stable levels on a subsequent session (Hussain et al., 2009b). When a fixed stimulus set is used, rapid improvements on this task occur within the first 200–300 trials, after which the gains are more gradual, with performance approaching ceiling after approximately 1700 practice trials (Hussain et al., 2009a,b). Here, we ask whether the early improvements within first 100 trials are stimulus specific, or whether they reflect only task-related learning. The emergence of stimulus specific learning after relatively few trials would suggest that the early and late phases of learning are not as distinct as previously assumed.

## Materials and Methods

### Subjects

Subjects were 32 undergraduate students at the University of Nottingham (mean age = 21 years). All had normal or corrected-to-normal vision.

### Apparatus and stimuli

Stimuli were generated in Matlab (v. 5.2) using the Psychophysics and Video Toolboxes (Brainard, 1997; Pelli, 1997), and displayed on a 21” Sony Trinitron monitor (1024 × 768 pixels) at a frame rate of 85 Hz. Average luminance was 40 cd/m^2^. Display luminance was measured with a PhotoResearch PR650 photometer, and the calibration data were used to build a 1779-element lookup table (Tyler et al., 1992). Customized computer software constructed the stimuli on each trial by selecting the appropriate luminance values from the calibrated lookup table and storing them in the display’s eight-bit lookup table.

The face stimuli were faces of five males and five female faces cropped to show only internal features (i.e., eyes, nose, and mouth). All of the faces had the same global amplitude spectrum (see Gold et al., 2004) for a more detailed description. The square patch within which the faces were embedded subtended 4.8° × 4.8° of visual angle, and the faces themselves subtended 2.5° × 3.7°. During the experiment, stimulus contrast was varied across trials using the method of constant stimuli. Seven levels of contrast were spaced equally on a logarithmic scale, and spanned a range that was sufficient to produce significant changes in performance in virtually all subjects. The stimuli were shown in one of three levels (low, medium, and high) of static two-dimensional Gaussian noise, created by sampling from Gaussian distributions with contrast variances of 0.001, 0.01, and 0.1. Hence, subjects viewed each stimulus at a signal-to-noise ratio that varied significantly across trials. Thus, there were 21 different stimulus conditions (seven contrast levels × three external noise levels).

### Procedure

All subjects performed the face identification task on two consecutive days. On Day 1, subjects performed 5 or 40 trials per condition (i.e., a total 105 or 840 trials per session). All subjects performed 40 trials per condition on Day 2. On Day 2, half the subjects from each group performed the task either with the same set of faces as on Day 1 or with a novel set of faces. Therefore, on Day 2, there were four groups of subjects that differed in terms of the number of trials per condition on Day 1 (5 vs. 40) and the type of stimuli seen on Day 2 (same vs. novel).

**Figure 1:**
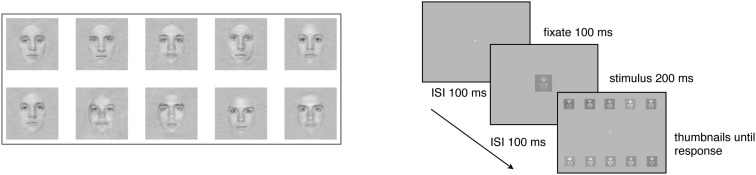
**Examples of stimuli used in the 10-AFC face identification tasks, and a schematic of the task**.

On each day, subjects were seated in a darkened room 114 cm away from the monitor. Viewing was binocular, and viewing position and distance were stabilized with an adjustable chin-rest. The experiment started after a 60 s period during which time the subject adapted to the average luminance of the display. A trial began with the presentation of a black, high-contrast fixation point (0.15° × 0.15°) in the center of the screen for 100 ms, followed by a face, selected randomly from the set of ten, in one of the 21 stimulus conditions, presented for 200 ms at the center of the screen. Since faces were randomly selected on each trial, each face was the target an average of 10 times for the 5-trials group, and an average of 84 times for the 40-trials group. After the face disappeared, the entire set of 10 faces was presented in a selection screen in two rows of five noiseless, high-contrast thumbnail images placed near the top and bottom of the display (see Figure 1). The location of each face in the response window was constant across trials and days. The subject’s task was to inspect the thumbnail images, and decide which one of the 10 faces had been presented during the trial by clicking on the chosen face with a computer mouse. Auditory feedback in the form of high-pitched (correct) and low-pitched (incorrect) tones informed the subject about the accuracy of each response, and the next trial began 1 s after presentation of the feedback.

## Results

**Figure 2:**
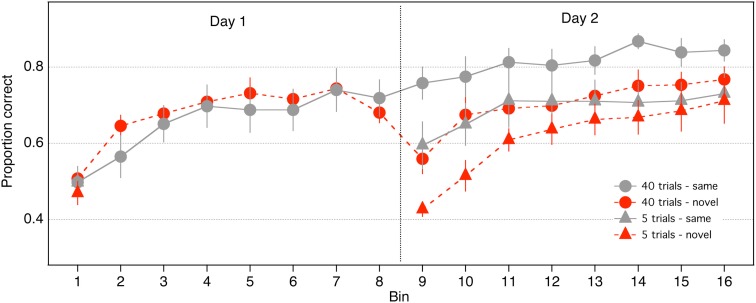
**Performance of the four groups on the face identification task over two consecutive days**.

Proportion correct was calculated in eight bins comprising 105 trials each, for each of the 2 days (16 bins across the 2 days; Figure 2). Preliminary analyses showed that noise did not interact with the results described below (*p* > 0.10 for all relevant interactions, or the direction of the effect did not vary as a function of noise), hence the 105 trials in each bin were collapsed across noise and contrast levels. The 40-trials groups performed 8 bins each on Days 1 and 2. The 5-trials groups performed one bin on Day 1 and 8 bins on Day 2.

We established that performance was equivalent across groups at the beginning of Day 1 through a one-way, between-groups ANOVA on accuracy in Bin 1, which showed that the effect of group was not significant [*F*(3,28) = 0.22, *p* = 0.88].

We first examined performance of the groups that received large amounts of practice (40 trials/condition). To assess within-session learning on Day 1 for the 40-trials groups, we conducted an 8 (bin) × 2 (stimulus) repeated measures ANOVA. A significant main effect of bin indicated that accuracy in the 40-trials groups increased across bins on Day 1 [*F*(7,98) = 23.58, *p* < 0.0001], demonstrating that within-session learning occurred in those groups. The effect of stimulus was not significant and did not interact with bin (*p* > 0.30). Accuracy of the 40-trials groups increased significantly from Day 1 to Day 2 [*F*(1,14) = 42.66, *p* < 0.0001], and a significant day × stimulus interaction indicated that the improvement was greater for the 40-same than the 40-novel group [*F*(1,14) = 22.19, *p* < 0.0001], which suggests that a significant component of the learning was stimulus specific. Consistent with this idea, performance of the 40-same group did not differ between Bin 8 and 9 [*t*(7) = 1.64, *p* = 0.1443], but accuracy of the 40-novel group dropped by 12% in Bin 9 relative to Bin 8 [*t*(7) = 4.76, *p* = 0.002]. Furthermore, accuracy of the 40-novel group in Bin 9 was 20% worse than accuracy of the 40-same group [*t*(13.92) = 3.38, *p* < 0.01]. In summary, these analyses confirm that significant stimulus specific learning occurred in the 40-trials groups.

Next, we examined if significant stimulus specific learning occurred with much smaller amounts of practice than had been examined previously (i.e., in the 5-trials group). We established that the 5-same group improved across Days [i.e., significant improvement from Bin 1 to Bin 9; Mean = 0.11; *t*(7) = 2.89, *p* = 0.02]. This finding replicates our previous work showing the positive effects of small amounts of practice on this task (Hussain et al., 2009b). A separate *t*-test indicated that performance of the 5-novel group did not change between Bin 1 and Bin 9 [*t*(7) = 1.11, *p* = 0.30]. Thus, small amounts of practice are effective in improving performance only if the stimuli remain fixed.

Figure 2 suggests that performance of the 5-novel group may have decreased from Bin 1 to Bin 9. To examine whether there was inhibition between bins for the 5-novel group (i.e., negative learning due to the transfer), we conducted an ANOVA on the difference between the first two successive bins for the novel versus same Groups (i.e., Bins 9 minus Bin 1 for the 5-trials groups and Bins 2 minus Bin 1 for the 40-trials group). A 2 × 2 mixed ANOVA (practice: 5 vs. 40 trials; stimulus: same vs. novel), revealed a significant main effect of practice [*F*(1, 28) = 5.50, *p* = 0.026], no significant effect of stimulus [*F*(1, 28) = 1.16, *p* = 0.28], and a significant interaction between practice and stimulus [*F*(1,28) = 10.99, *p* = 0.002]. The main effect of practice confirms an overall larger improvement for the 40 trials than the 5-trials group (which is not surprising because the 5-novel group did not increase from Bin 1 to Bin 9). The significant interaction suggests that the effect of stimulus depended on the amount of practice. We decomposed this interaction through separate t-tests examining the amount of improvement for each group. The improvement of the 5-same, 40-same, and 40-novel groups did not differ from each other (*p* > 0.05), but all groups differed from the 5-novel group (*p* < 0.05). As mentioned above, the change in performance of the 5-novel group between Bins 1 and 9 did not significantly differ from zero. These analyses suggest that (1) there was positive improvement for all groups that saw the same stimuli on successive bins, whereas (2) there was no change (no increase, and no inhibition) in performance for the group that transferred to novel stimuli.

A 2 (stimulus: same vs novel) × 2 (practice: 5 vs 40 trials per condition) between-subjects ANOVA was computed on accuracy measured in the first bin on Day 2 (i.e., Bin 9). There were significant main effects of stimulus [*F*(1,28) = 17.3753, *p* < 0.001] and practice [*F*(1,28) = 11.2651, *p* < 0.01], but the stimulus × practice interaction was not significant [*F*(1,28) = 0.12, *p* < 0.73]. Furthermore, the fact that the *F* value for the interaction was less than one means that the best estimate of the interaction’s effect size is zero (Kirk, 1995). The absence of a significant interaction suggests that the advantage of same over novel stimuli in Bin 9 did not depend on the amount of practice on Day 1 (see Figure 2). Indeed, as was the case with the 40-trials groups, accuracy of the 5-novel group in Bin 9 was 17% worse than that of the 5-same group. In other words, stimulus specific learning, as indexed by performance on Bin 9, was equivalent for the 40-trials and 5-trials groups. Another way of stating this result is that large amounts of practice (i.e., practice trials later in the time course of learning) had equivalent benefits for same and novel stimuli. The effects of late practice trials, rather than being confined to stimuli practiced in the first session, extended to novel stimuli viewed in the second session.

Learning on Day 2 was analyzed with an 8 (bin) × 2 (practice trials) × 2 (stimulus) ANOVA. There was a significant main effect of bin [*F*(7,196) = 26.1031, *p* < 0.0001], reflecting the fact that accuracy generally increased with bin number. There was a significant main effect of practice [*F*(1,28) = 6.7916, *p* < 0.015], indicating that accuracy was greater in the 40-trials groups. There was also a significant main effect of stimulus, indicating that accuracy of the same stimulus groups was greater than that of the novel stimulus groups [*F*(1,28) = 5.3681, *p* < 0.03]. Figure 2 suggests that on Day 2 the novel stimulus groups showed more improvement than the same stimulus groups. This was confirmed by a significant stimulus × bin interaction [*F*(7,196) = 3.49, *p* < 0.01]. None of the other interactions were significant.

Finally, Hussain et al. (2009b) reported that the effect of practice depended primarily on the number of practice trials and not on how those trials were distributed across days, and therefore response accuracy of the 5-same group in Bin 9 (on Day 2) ought to be the same as the performance of the two 40-groups in Bin 2 (on Day 1). To test this prediction, we performed an ANOVA on response accuracy measured in Bin 9 for the 5-same group and Bin 2 for the 40-same and 40-novel groups: the effect of group was not significant [*F*(2,21) = 0.64, *p* < 0.54]. Next, we performed a 3 (group) × 7 (bin) ANOVA on response accuracy measured in Bins 2–8 in the 40-same and 40–novel groups and Bins 9–15 for the 5-same group. The main effect of bin was significant [*F*(6,126) = 12.42, *p* < 0.001], but the main effect of group [*F*(2,21) = 0.065, *p* < 0.94] and the group × bin interaction [*F*(12,126) = 0.95, *p* < 0.50] were not. These analyses suggest that the performance of the 5-same group on Day 2 did not differ from the performance of the 40-trials groups on Day 1.

## Discussion

Even small amounts of practice produced stimulus specific learning in a 10-AFC identification task: response accuracy measured on Day 2 was significantly greater in subjects shown a familiar set of faces (i.e., those shown on Day 1) compared to subjects shown a novel set of faces. The degree of specificity, as indexed by the difference in performance obtained with familiar and novel stimuli at test, did not depend on whether subjects received 105 or 840 trials of practice the preceding day. Furthermore, task-related learning was not confined to early practice trials: accuracy for novel faces was higher for the group that received more practice on Day 1, reflecting the contribution of late practice trials to non-specific improvements (see also Wright et al., 2010). Hence, precise stimulus properties were encoded early, and procedural improvements distributed throughout the time course of learning of the task.

There is some evidence that performance in some perceptual and motor tasks depends not only on the number of practice trials but also on the distribution of practice trials across time (Savion-Lemieux and Penhune, 2005). These effects have been interpreted as evidence for a consolidation process that may be influenced by sleep (Censor et al., 2006). If sleep had a significant effect on perceptual learning in our task, we would expect to find a difference between performance of the 5-same group on Day 2 and the 40-trials groups on Day 1 (i.e., better performance of the 5-same group than the 40-trials groups due to the overnight interval). However, our analyses did not support this prediction. Instead, our results suggest that the effect of sleep on perceptual learning of faces is small, and that performance depends primarily on the number of practice trials rather than the way those trials are distributed across days (Aberg et al., 2009; Hussain et al., 2009b).

A recent study (Jeter et al., 2010) found non-specific learning of Gabor orientation discrimination after relatively small amounts of practice, and increasing stimulus specificity thereafter: two practice blocks comprising 1248 trials yielded generalization to an untrained location and orientation, whereas 12 practice blocks comprising 7488 trials produced location- and orientation-specific learning. For a Chevron discrimination task, Aberg et al. (2009) found that learning was specific to orientation when intense practice (≈800 trials per session) was given, but transferred to the orthogonal orientation with fewer practice trials per session (≈400 trials), similar to the results of Jeter et al. Although the current experiment used an amount of practice (105 trials) that was considerably less than that used by Jeter et al and Aberg et al the learning we observed was stimulus specific. The discrepancy between studies may reflect differences in the generalized dimension (location and orientation versus identity), or the stimulus and task complexity, both of which varied across studies. Jeter et al characterized their task as a high precision task, but stimulus specific learning can occur in far fewer trials for other similarly precise tasks (e.g., hyperacuity; Poggio et al., 1992), suggesting that task precision does not predict the onset of stimulus specificity in learning. Other factors such as task difficulty (Ahissar and Hochstein, 1997), task precision (Jeter et al., 2009), sleep-dependent consolidation (Karni et al., 1994), variations of the training method (Xiao et al., 2008), and variability of the stimulus set (Hussain et al., 2012) have all been shown to affect the amount (but not onset) of stimulus specificity after practice on a particular task. Some combination of these factors may also account for why the onset of specificity is early for particular tasks, and delayed for others.

Face identification is an example of a high-level, behaviorally relevant visual task in which rapid learning might be advantageous, but rapid stimulus specific learning on the time-scale of 200 trials has been found for rudimentary visual judgments such as grating discrimination (Fiorentini and Berardi, 1980) and hyperacuity (Poggio et al., 1992), suggesting that similar computational rules might underlie the learning of simple and complex spatial patterns. The current data are consistent with a single-process functional architecture that has been described for learning of orientation signals (Dosher and Lu, 2007), and for the abrupt, insight-like learning of larger spatial patterns (Rubin et al., 1997), but not with models that differentially weight task and signal at successive stages in the time course of learning (Jeter et al., 2010).

## Conflict of Interest Statement

The authors declare that the research was conducted in the absence of any commercial or financial relationships that could be construed as a potential conflict of interest.
